# When the Aortic Root Strikes the Conduction System: Lessons From a Rare Case

**DOI:** 10.1016/j.jaccas.2026.108240

**Published:** 2026-05-13

**Authors:** Edoardo Zancanaro, Tommaso Hinna Danesi

**Affiliations:** Division of Cardiac Surgery, Brigham and Women's Hospital, Harvard Medical School, Boston, Massachusetts, USA

**Keywords:** aorta, aortic valve, bradycardia, cardiac pacemaker, computed tomography, echocardiography, electrocardiogram

**Figure:**
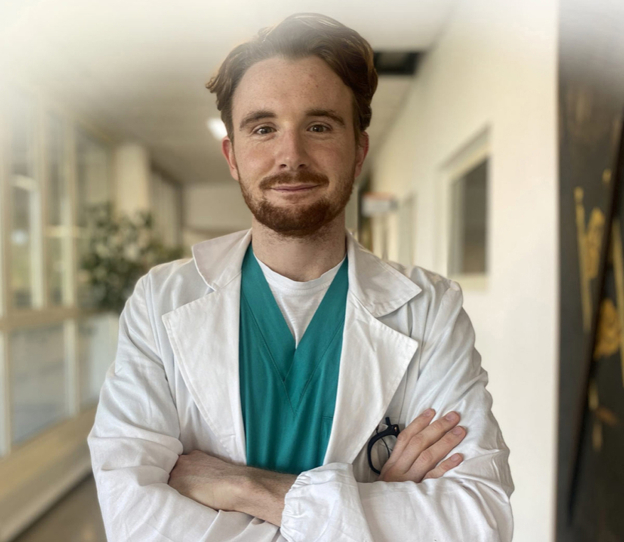

**Visual Summary:**
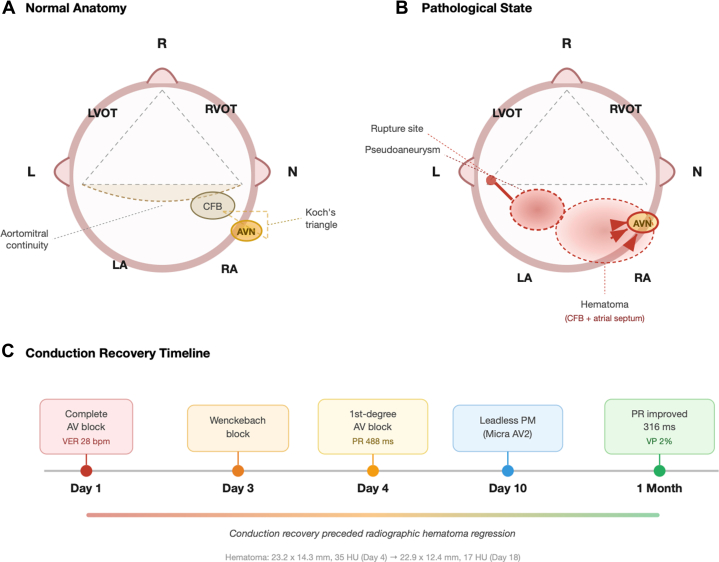
Mechanism and Recovery of AV Block Due to Left Coronary Aortic Sinus Rupture (A) Normal short-axis anatomy of the aortic root. The 3 sinuses of Valsalva (R, L, and N), aortomitral continuity, CFB, and AVN within Koch's triangle are shown. (B) Pathologic state: rupture of the left coronary sinus created a pseudoaneurysm and hematoma extending into the CFB and atrial septum, mechanically compressing the AVN (red arrows). (C) Timeline of stepwise conduction recovery, which preceded radiographic evidence of hematoma regression. AV = atrioventricular; AVN = atrioventricular node; CFB = central fibrous body; LA = left atrium; LVOT = left ventricular outflow tract; RA = right atrium; RVOT = right ventricular outflow tract.

The cardiac conduction system, despite its critical functional importance, is remarkably vulnerable to structural insults. Nestled within the central fibrous body at the confluence of the aortic root, the mitral annulus, the tricuspid annulus, and the atrial septum, the atrioventricular (AV) node and the bundle of His occupy a precarious anatomic neighborhood. This spatial relationship is well appreciated in the context of surgical and transcatheter aortic valve interventions, where conduction disturbances remain among the most frequent complications. Indeed, Handa et al^7^ recently demonstrated that variations in aortomitral positional anatomy, including aortic root rotation, influence the risk of postoperative atrioventricular conduction disorders, reinforcing how intimately the conduction system's fate is tied to its structural surroundings. However, the spontaneous disruption of these same structures—and the consequent impact on AV conduction—remains an underrecognized clinical entity.

In this issue of *JACC: Case Reports*, Kawase et al^1^ present a compelling case of an 85-year-old woman who developed complete AV block secondary to spontaneous rupture of the left coronary aortic sinus. The ensuing pseudoaneurysm and hematoma extended through the aortomitral continuity into the central fibrous body and atrial septum, mechanically compressing the AV node region within the triangle of Koch.^2^ What makes this case particularly instructive is not only its rarity, reportedly the first instance of AV block from left coronary sinus rupture, but the meticulous anatomic documentation and the clearly demonstrated reversibility of the conduction disturbance as the hematoma regressed.

The anatomic precision of this report deserves emphasis. The authors used contrast-enhanced computed tomography with volume-rendered reconstructions to map the spatial relationships among the pseudoaneurysm, the hematoma, and the conduction system landmarks. The demonstration of abnormal bulging of the floor of the triangle of Koch, demarcated by the tendon of Todaro, the coronary sinus ostium, and the septal tricuspid leaflet attachment, provides a textbook-quality illustration of how extracardiac pathology can impinge on the compact AV node. This level of anatomic detail transforms the case from a clinical curiosity into a teaching resource.

The temporal correlation between conduction recovery and hematoma evolution is equally noteworthy. AV conduction began improving on day 3, progressing from complete heart block to a Wenckebach pattern, then to first-degree AV block by day 4, while computed tomography evidence of hematoma regression only became apparent by day 18. The authors astutely interpret this discrepancy through the lens of the wound-healing cascade: the decrease in compartment pressure from the inflammatory resorption phase precedes measurable volumetric reduction. This pathophysiological reasoning is sound and mirrors observations in other clinical settings where neural or conductive function recovers before structural resolution is radiographically apparent.^4^

Several clinical lessons emerge from this case. First, the differential diagnosis of acute complete AV block must extend beyond the usual suspects of ischemia, drug toxicity, and degenerative fibrosis to include mechanical compression from aortic root pathology. Although AV block from rupture of the right or noncoronary sinuses of Valsalva has been described,^3^^,^^8^ and unruptured aneurysms of the noncoronary sinus may similarly compress the conduction system,^9^ the left coronary sinus is anatomically more remote from the conduction system, making this presentation unexpected. The authors appropriately considered infective endocarditis with annular abscess, antineutrophil cytoplasmic antibody–associated vasculitis, penetrating atherosclerotic ulcer, and localized aortic dissection as alternative mechanisms, a differential that reflects the nuanced thinking required in such cases.

Second, this case illustrates the importance of multimodality imaging in elucidating the mechanism of conduction disturbances. Echocardiography identified the abnormal cavity between the aortic sinuses, but contrast-enhanced computed tomography was essential for characterizing the fistulous tract, quantifying the hematoma, and mapping its extension into the conduction system territory. In an era of increasingly sophisticated cardiac imaging, this case reminds us that the most clinically meaningful images are those interpreted through a deep understanding of cardiac anatomy (Table 1).

**Table 1:** Summary of Published Cases of AV Block Associated With Sinus of Valsalva Pathology

First Author	Sinus Involved	Mechanism	Conduction Disturbance	Reversibility	Management
Bocchino et al^3^	Right	Rupture, septal dissection	Transient complete AV block	Yes	Conservative
Sato et al^9^	Noncoronary	Unruptured aneurysm, thrombus	Complete AV block	No	PM implantation
Yi et al^8^	Right	Rupture, septal dissection	Third-degree AV block	Yes (postsurgery)	AVR + aneurysm repair
Kawase et al^1^	Left	Rupture, hematoma via CFB	Complete AV block first degree	Yes (spontaneous)	Conservative + leadless PM

AV = atrioventricular; AVR = aortic valve replacement; CFB = central fibrous body; PM = pacemaker.

Third, the management decisions in this case reflect the complexity of treating older, frail patients with acute cardiac pathology. With a Clinical Frailty Scale score of 7 and an Society of Thoracic Surgery-predicted mortality of nearly 12%, the heart team appropriately deemed surgical intervention prohibitive. The subsequent decision to implant a leadless pacemaker for intermittent residual AV block was pragmatic, balancing the low anticipated pacing burden against the patient's functional status and life expectancy. The 2% ventricular pacing percentage at 1-month follow-up validated this approach.^5^

Finally, this case invites broader reflection on the intersection of structural heart disease and electrophysiology. As our understanding of the aortic root's complex 3-dimensional relationships with the conduction system deepens, driven in part by the explosive growth of transcatheter interventions, we should remain vigilant for spontaneous pathology affecting these same structures. The central fibrous body is not merely a passive scaffold; it is a dynamic anatomic crossroads where vascular, valvular, and electrical pathways converge. Pathology in one domain can affect the others.^6^

Kawase et al^1^ have provided a meticulously documented case that enriches our understanding of the mechanical causes of AV block. Their work underscores a fundamental principle: when confronted with an unexplained conduction disturbance, always look to the anatomy. The conduction system may be invisible on standard imaging, but the structures that surround and protect it are not—and when those structures fail, the electrical consequences can be both dramatic and, as this case beautifully demonstrates, reversible.

## Funding Support and Author Disclosures

The authors have reported that they have no relationships relevant to the contents of this paper to disclose.

## References

[1] Kawase K., Nakajima K., Miyazaki Y. (2026). Reversible complete atrioventricular block due to rupture of the left coronary aortic sinus. JACC Case Rep.

[2] Mori S., Fukuda T., Miwa K. (2021). Anatomic relations between the aortic root and the cardiac conduction system: relevance to transcatheter aortic valve implantation. J Cardiovasc Electrophysiol.

[3] Bocchino P.P., Fasano R., Fortuni F. (2021). Transient complete atrioventricular block due to rupture of the right sinus of Valsalva. Circ Cardiovasc Imaging.

[4] Kopelman H.A., Graham B.S., Forman M.B. (1986). Myocardial abscess with complete heart block complicating anaerobic infective endocarditis. Br Heart J.

[5] Piazza N., de Jaegere P., Schultz C. (2008). Anatomy of the aortic valvar complex and its implications for transcatheter implantation of the aortic valve. Circ Cardiovasc Interv.

[6] Hamdan A., Guetta V., Klempfner R. (2015). Inverse relationship between membranous septal length and the risk of atrioventricular block in patients undergoing transcatheter aortic valve implantation. JACC Cardiovasc Interv.

[7] Handa K., Kawamura M., Yoshioka D. (2024). Impact of the aortomitral positional anatomy on atrioventricular conduction disorder following mitral valve surgery. J Am Heart Assoc.

[8] Yi S.Y., Zhou C., Feng J. (2022). Rupture of the right sinus of Valsalva aneurysm and formation of ventricular septal dissection and third-degree atrioventricular block: a case report. SAGE Open Med Case Rep.

[9] Sato W., Seki K., Yamamoto H., Watanabe H. (2021). Thrombosed sinus of Valsalva aneurysm masquerading as a cardiac tumour: a case report. Eur Heart J Case Rep.

